# Spindle Assembly Checkpoint Competency Determines Sensitivity to KIF18A Inhibition in Small-Cell Lung Cancer

**DOI:** 10.64898/2026.07.30.741836

**Published:** 2026-07-31

**Authors:** Chiori Tabe, Rajesh Kumar, Yue Huang, Michael J. Kruhlak, Ajit K. Sharma, Roshan L. Shrestha, Anish Thomas

## Abstract

**Background:**

Small-cell lung cancer (SCLC) is characterized by pervasive chromosomal instability (CIN) and remains largely refractory to targeted therapies. KIF18A, a motor protein that regulates chromosome alignment during mitosis, has emerged as a selective dependency in CIN-high tumors. Whether this dependency extends to SCLC, a prototypical CIN-high cancer, has not been established, and biomarkers predicting response to KIF18A inhibition, currently in clinical trials, are lacking.

**Methods:**

We integrated analyses of patient tumor datasets, neuroendocrine (NE) and non- NE SCLC cell lines, and functional perturbation models to define the determinants of response to KIF18A inhibition. Chromosomal instability metrics, transcriptional programs, mitotic dynamics, and spindle assembly checkpoint (SAC) function were assessed using genomic profiling, live-cell imaging, genetic perturbation, and pharmacologic inhibition.

**Results:**

KIF18A expression was elevated in SCLC tumors and correlated with CIN-associated transcriptional programs, proliferative markers, and NE status; however, these features did not predict sensitivity to KIF18A inhibition. Instead, response was determined by the functional integrity of the SAC. SAC-proficient SCLC cells underwent sustained mitotic arrest followed by apoptotic cell death upon KIF18A inhibition, whereas SAC-defective cells failed to maintain checkpoint activation and survived. Mechanistically, resistant cells exhibited impaired kinetochore recruitment of core SAC components, including MAD1 and BUBR1. Importantly, transient induction of acute CIN through MPS1 inhibition partially restored sensitivity to KIF18A inhibition in resistant models.

**Conclusions:**

This study provides the first mechanistic characterization of KIF18A dependency in SCLC, identifying SAC competency as the primary determinant of response. These findings establish a biologically informed framework for patient stratification and rational combination strategies.

**Translational Relevance:**

Small-cell lung cancer (SCLC) is an aggressive malignancy with few effective targeted therapies and marked chromosomal instability. KIF18A has emerged as a potential therapeutic target in genomically unstable cancers, but biomarkers predicting response to KIF18A inhibition are lacking. We demonstrate that sensitivity to KIF18A inhibition in SCLC is determined not by KIF18A expression, neuroendocrine subtype, or baseline chromosomal instability, but by the functional integrity of the spindle assembly checkpoint (SAC). SCLC cells with intact SAC signaling undergo sustained mitotic arrest and apoptosis upon KIF18A inhibition, whereas SAC-defective cells bypass checkpoint activation and survive aberrant mitosis. Notably, transient induction of acute chromosomal instability through MPS1 inhibition partially restores sensitivity in resistant models. Together, these findings identify mitotic checkpoint competency as a mechanistic determinant and candidate predictive biomarker for KIF18A-targeted therapies, providing a biologically informed framework for patient stratification and rational combination strategies relevant to ongoing KIF18A inhibitor clinical trials.

## Full Text Availability

The license terms selected by the author(s) for this preprint version do not permit archiving in PMC. The full text is available from the preprint server.

